# Supplementary material for: Predicting 30-day and 1-year mortality in heart failure with preserved ejection fraction (HFpEF)
Source: PLoS One. 2025 Nov 14;20(11):e0336809. doi: 10.1371/journal.pone.0336809 (PMC12617840; doi:10.1371/journal.pone.0336809)
Supplement: S2 Table — (PDF) [file pone.0336809.s002.pdf]

S2 Table. HFpEF related ICD codes and their frequencies.

long_title	icd_code	frequency
Acute diastolic (congestive) heart failure	I5031	66
Acute diastolic heart failure	42831	292
Acute on chronic diastolic (congestive) heart failure	I5033	584
Acute on chronic diastolic heart failure	42833	2138
Chronic diastolic (congestive) heart failure	I5032	7
Chronic diastolic heart failure	42832	83
Diastolic heart failure, unspecified	42830	58
Unspecified diastolic (congestive) heart failure	I5030	7
