# Supplementary material for: Predicting 30-day and 1-year mortality in heart failure with preserved ejection fraction (HFpEF)
Source: PLoS One. 2025 Nov 14;20(11):e0336809. doi: 10.1371/journal.pone.0336809 (PMC12617840; doi:10.1371/journal.pone.0336809)
Supplement: S3 Table — (PDF) [file pone.0336809.s003.pdf]

S3 Table. Features, outcomes and their data types.

Data Type	Variable Name	Original Variable Name
target	death_30_days	death_within_30_days
target	death_1_year	death_within_1_year
numeric	age_admission	age_admission
numeric	temperature	temperature
numeric	heart_rate	heartrate
numeric	oxygen_saturation	o2sat
numeric	systolic_bp	sbp
numeric	bmi	BMI (kg/m2)
numeric	bicarbonate	Bicarbonate_mEq/L
numeric	creatinine	Creatinine_mg/dL
numeric	hemoglobin	Hemoglobin_g/dL
numeric	inr	INR(PT)_NA
numeric	platelet_count	Platelet Count_K/uL
numeric	potassium	Potassium_mEq/L
numeric	wbc_count	WBC Count_K/uL
numeric	sodium	Sodium_mEq/L
numeric	ntprobnp	NTproBNP_pg/mL
numeric	troponin	Troponin T
binary	gender	gender
binary	acute_myocardial_infarction	AMI (Acute Myocardial)
binary	peripheral_vascular_disease	PVD (Peripheral Vascular)
binary	cerebrovascular_disease	CEVD (Cerebrovascular)
binary	dementia	Dementia
binary	chronic_obstructive_pulmonary_disease	COPD (Chronic Obstructive Pulmonary)
binary	rheumatoid_disease	Rheumatoid Disease
binary	peptic_ulcer_disease	PUD (Peptic Ulcer)
binary	mild_liver_disease	Mild LD (Liver)
binary	diabetes	Diabetes
binary	diabetes_complications	Diabetes + Complications
binary	hemiplegia_paraplegia	HP/PAPL (Hemiplegia or Paraplegia)
binary	renal_disease	RD (Renal)

binary	cancer	Cancer
binary	moderate_severe_liver_disease	Moderate/Severe LD (Liver)
binary	metastatic_cancer	Metastatic Cancer
binary	HT	hypertension
binary	CAD	coronary artery disease
binary	PH	pulmonary hypertension
binary	AF	atrial fibrillation
