# Supplementary material for: Predicting 30-day and 1-year mortality in heart failure with preserved ejection fraction (HFpEF)
Source: PLoS One. 2025 Nov 14;20(11):e0336809. doi: 10.1371/journal.pone.0336809 (PMC12617840; doi:10.1371/journal.pone.0336809)
Supplement: S4 Table — (PDF) [file pone.0336809.s004.pdf]

S4 Table. Missing values (count and %) of features.

**Feature**	**Count (n)**	**Percentage (%)**
troponin	1953	60.37
bmi	1409	43.55
temperature	1131	34.96
oxygen_saturation	934	28.87
inr	823	25.44
heart_rate	796	24.61
systolic_bp	758	23.43
ntprobnp	535	16.54
sodium	141	4.36
potassium	101	3.12
wbc_count	74	2.29
platelet_count	68	2.10
hemoglobin	59	1.82
bicarbonate	55	1.70
creatinine	37	1.14
