# Supplementary figures and images for: Predicting 30-day and 1-year mortality in heart failure with preserved ejection fraction (HFpEF)
Source: PLoS One. 2025 Nov 14;20(11):e0336809. doi: 10.1371/journal.pone.0336809 (PMC12617840; doi:10.1371/journal.pone.0336809)

**S1 Fig. Correlation matrix for continuous variables in (A) 30-day and (B) 1-year mortality.**

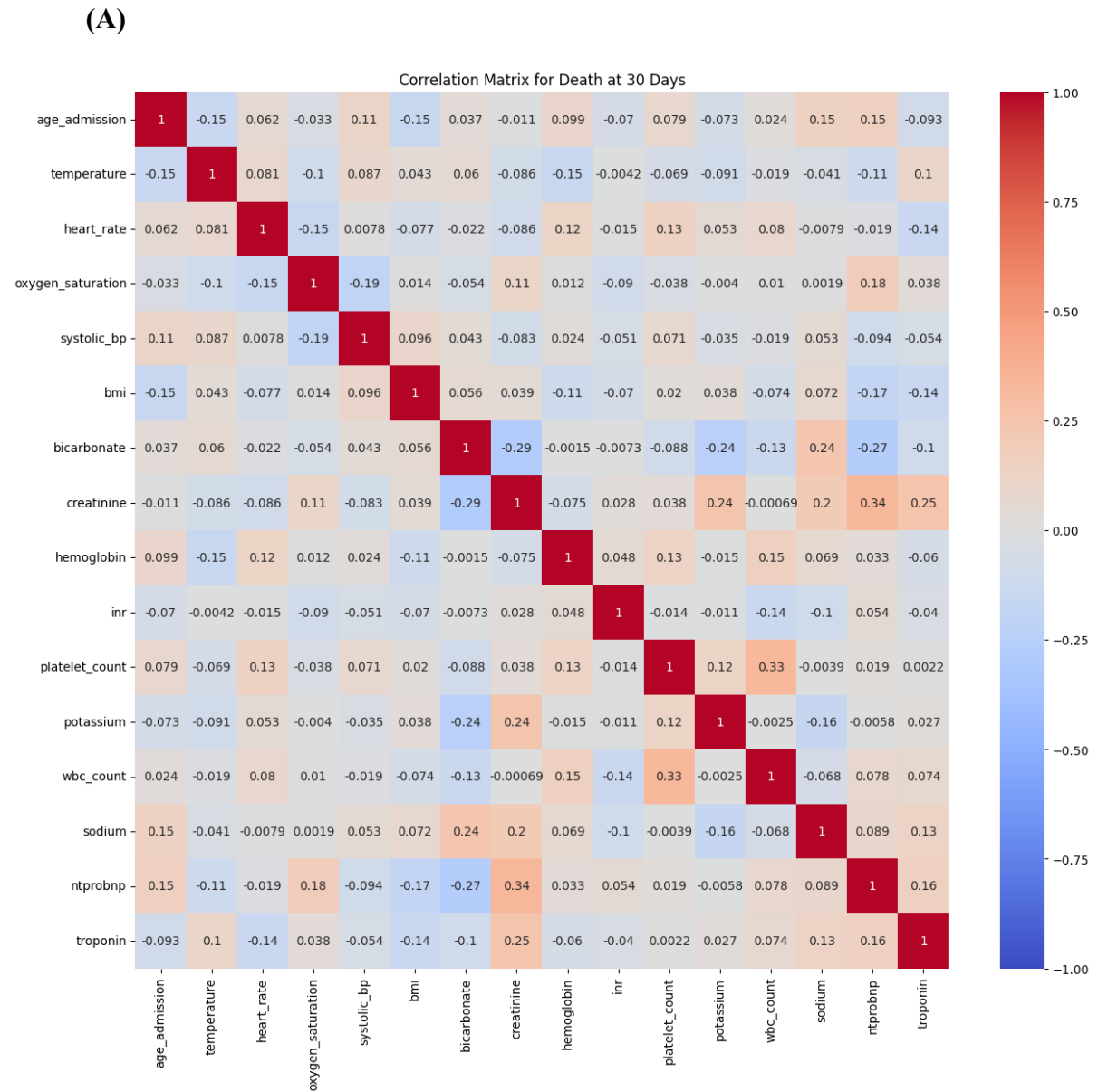

(B)

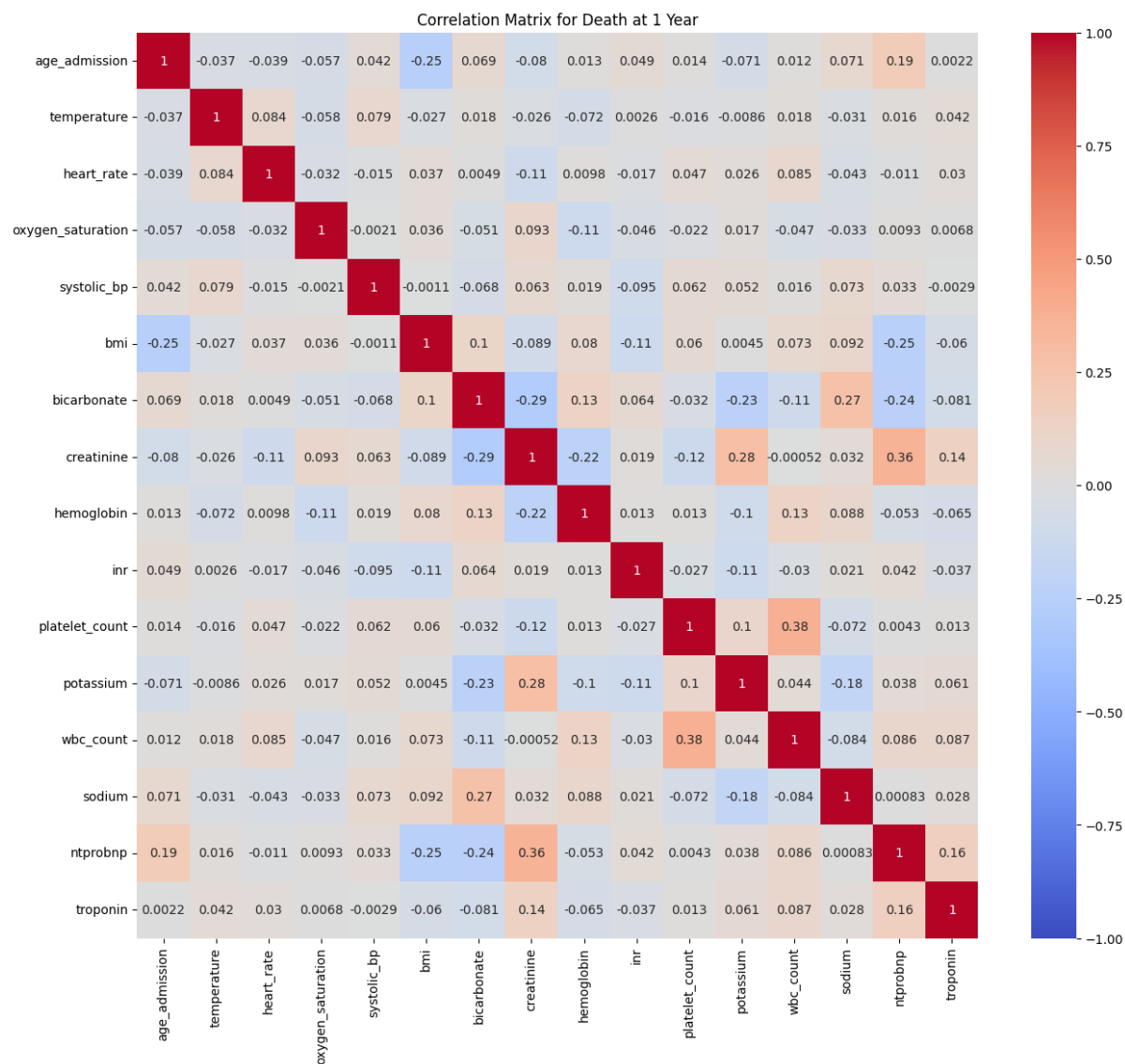

Supplement: S1 Fig — (PDF) [file pone.0336809.s005.pdf]

**S2 Fig. Mutual information (MI) analysis for (A) 30-day and (B) 1-year mortality.**

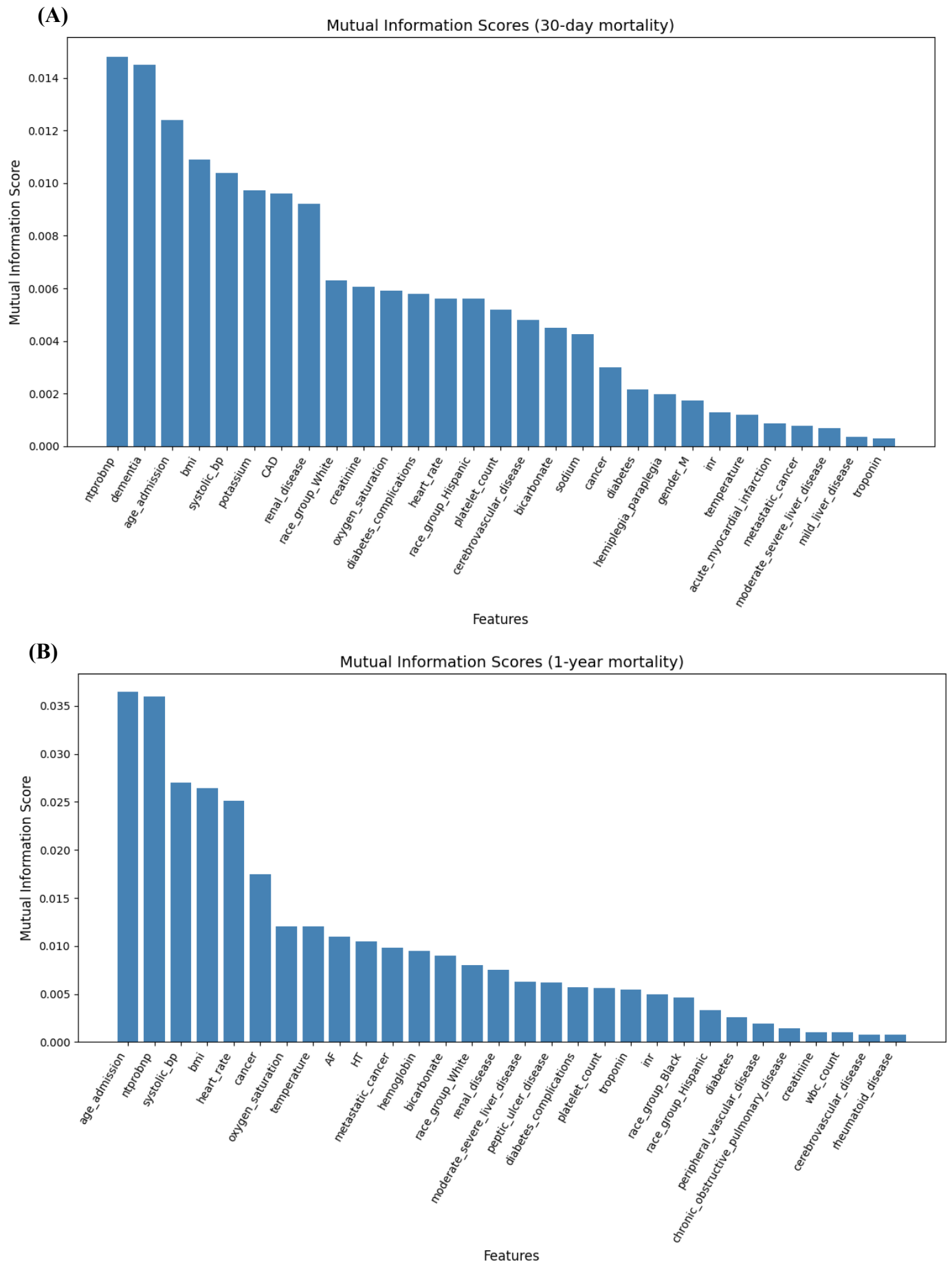

Supplement: S2 Fig — (PDF) [file pone.0336809.s006.pdf]

**S3 Fig. Precision-recall curves for (a) 30-day and (b) 1-year mortality.**

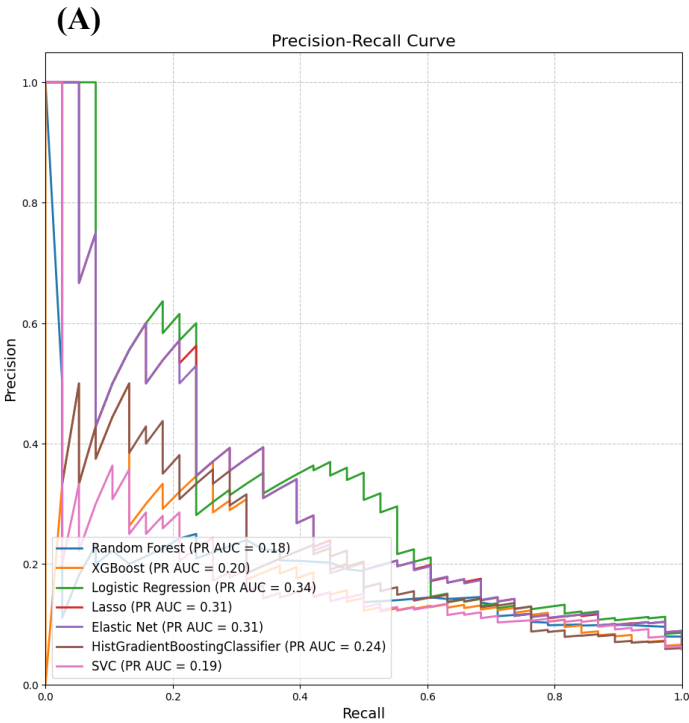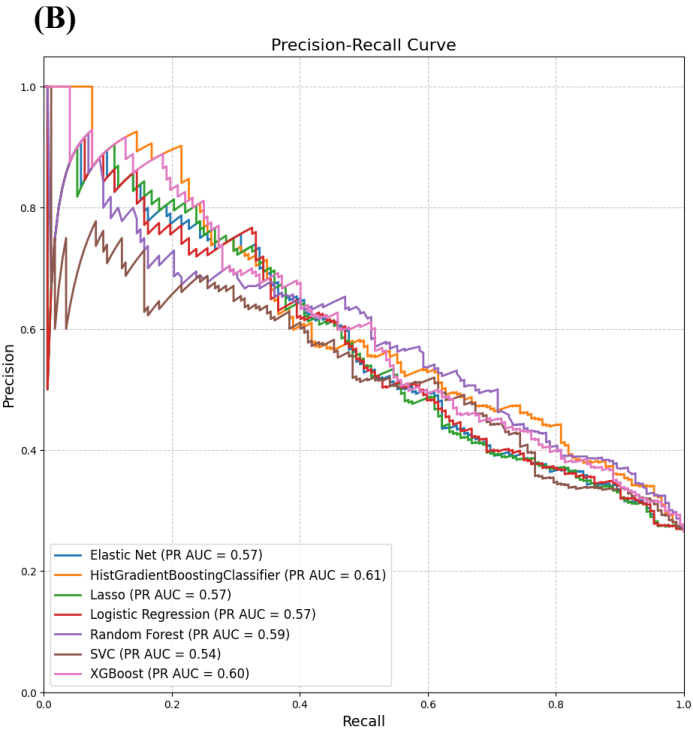

Supplement: S3 Fig — (PDF) [file pone.0336809.s007.pdf]

**S4 Fig. Calibration curves for (A) 30-day and (B) 1-year mortality outcomes.**

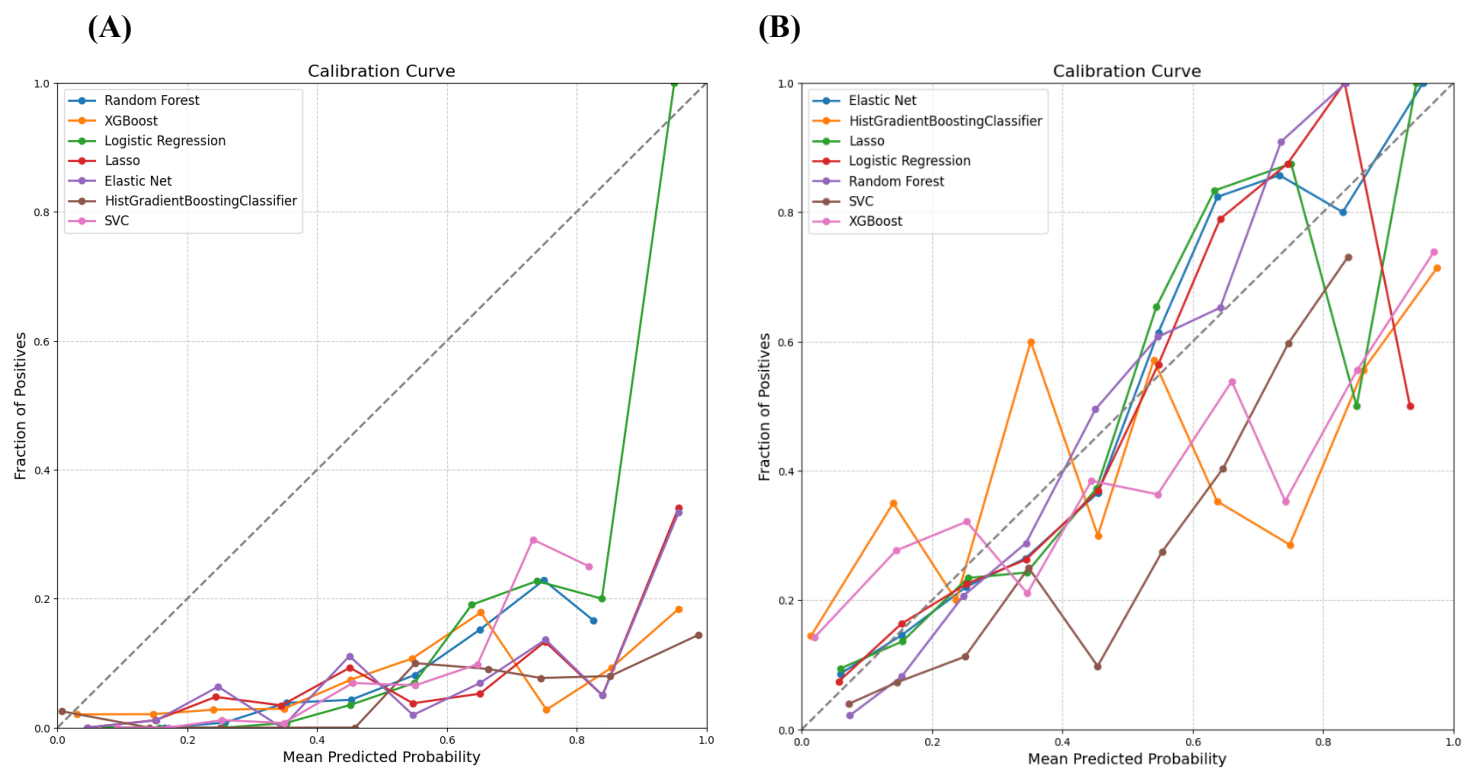

Supplement: S4 Fig — (PDF) [file pone.0336809.s008.pdf]
