# Supplementary material for: Predicting 30-day and 1-year mortality in heart failure with preserved ejection fraction (HFpEF)
Source: PLoS One. 2025 Nov 14;20(11):e0336809. doi: 10.1371/journal.pone.0336809 (PMC12617840; doi:10.1371/journal.pone.0336809)

S5 Fig. SHAP bar plots for (A) 30-day mortality - logistic regression model and (B) 1-year mortality - HGBC model.

(A)

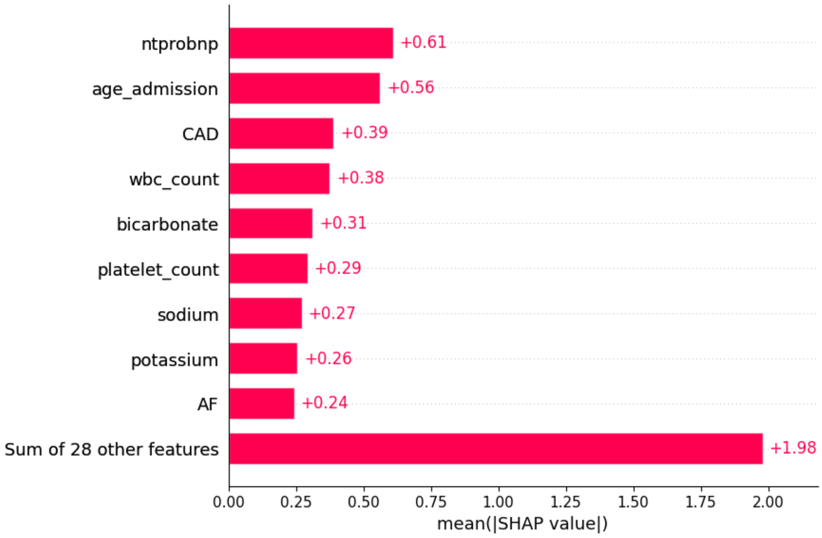

(B)

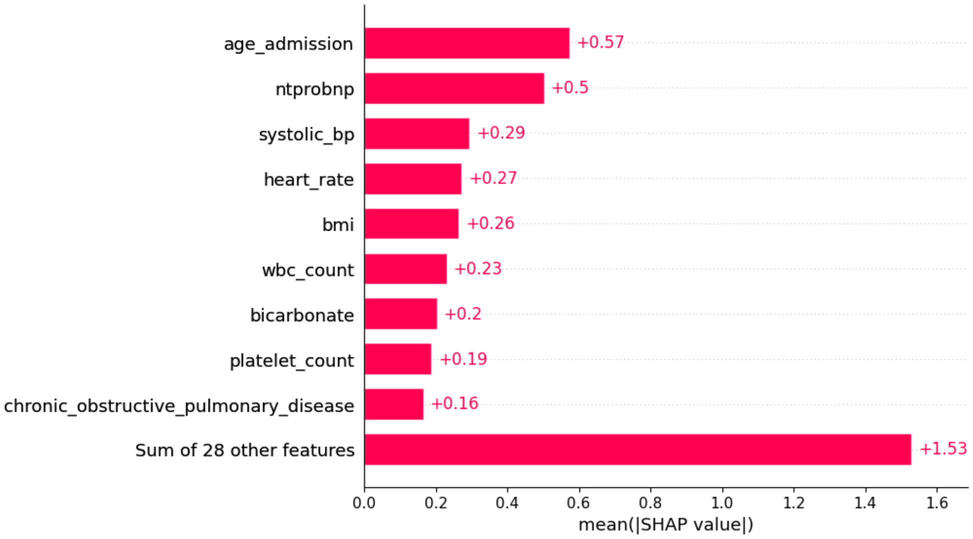

Supplement: S5 Fig — (PDF) [file pone.0336809.s009.pdf]
