# Supplementary material for: Predicting 30-day and 1-year mortality in heart failure with preserved ejection fraction (HFpEF)
Source: PLoS One. 2025 Nov 14;20(11):e0336809. doi: 10.1371/journal.pone.0336809 (PMC12617840; doi:10.1371/journal.pone.0336809)
Supplement: Attachment — Submitted filename: Response to Reviewers.docx [file pone.0336809.s010.docx]

August 22^nd^, 2025

To

Dr. Emily Chenette

Editor-in-Chief

*PLOS One*
1875 Mission St., Suite 103 #188

San Francisco, CA 94103

Sub: Rebuttal Letter for Manuscript #PONE-D-25-18607

Dear Dr. Chenette and Editor Dr. Hage,

Thank you for the opportunity to revise and resubmit our manuscript titled “Predicting 30-Day and 1-Year Mortality in Heart Failure with Preserved Ejection Fraction (HFpEF)” to *Plos One*. We thank the reviewers of *Plos One* for their detailed comments on the manuscript and have edited the manuscript to address their concerns, which has improved the research paper. The line-by-line responses to the reviewer comments are included below.

*Reviewer #1:*

*This study investigates prognostic models of 30-days and 1-year mortality in HFpEF patients using EHR data sources. The authors demonstrate that logistic regression model predicted 30-day mortality whereas Random Forest and Histogram-based Gradient Boosting Classifier predicted 1-year mortality. Age and NT-proBNP were the strongest predictors in SHapley Additive exPlanations analyses for both outcomes.
Overall, the study design is adequate, and the paper is clearly written. The presented results seem valid and reliable. The analyses are performed in real-world data.

Major comment and Clinical utility:
Rigorously performed analyses demonstrating the use of advanced models. These can be used as algorithms assisting clinicians if based on few clinically available variables and highlighting certain variables of importance. The present data shows the importance of age and NPs as prognostic predictors. Can be questioned if this information adds to the clinicians’ decision and risk assessment beyond what we already know.*

Response: Thank you for the comment. As the Reviewer points out, some of the identified predictors such as age and NT-proBNP have been noted previously in literature to predict outcomes in HFpEF. However, the prior existing risk models in HFpEF have been derived from registry or trial data and are for ambulatory populations. The novelty of our study is the use of real-world EHR data from a large health system and the being model specific to the hospitalized population. Thus, although there is some commonality of variables identified with prior models, the strength of association with the outcomes may differ in this treatment setting. Further, the incomplete nature of EHR data also can change association of variables. An EHR derived model is likely to perform better in a patient-facing EHR setting. However, despite these advantages further validation of our model is needed.

*Specific points:

Abstract:
-
Background:
Misspelling at row 68 (HER).*

Response: Thank you for pointing out this error, we have corrected it in the revision to read as ‘EHR’.

*Methods:
Regarding the selection of patients based on ICD codes. Were patients with recorded LVEF =<40% in stable condition or during a later hospitalization excluded?
Was data extracted for the full time period 2008-2019?*

Response: Thank you for this question. To clarify, the identification of HFpEF in this study was primarily made using ICD-9 and ICD-10 codes. However, as this is a code-based identification we wanted to enhance the validity of this method of identification by extracting ventricular function information from the associated clinical notes using regular expression.

Among the 3235 patients, 3146 (97.3%) had clinical notes available to analyze. Overall, 58.4% had an LVEF measurement value reported in the clinical notes of whom 94.0% had an LVEF value ≥ 50%, and 2.5% had an LVEF between 45 to 50%. Another 18.6% encounters had a qualitative mention of LVEF, of which 94.0% indicated the LVEF was ‘normal’ or ‘preserved’. The remaining 23% of the cohort had no quantitative or qualitative mention of ventricular information in their clinical notes.

Given that among the 77% encounters with either a quantitative or qualitative mention of ventricular mention, >97% had a documented preserved LVEF we concluded that this method of ICD code-based identification of HFpEF that we used in our study is a valid method and has sufficient positive predictive value. This validation information will also be useful for future projects by other researchers that use ICD codes to identify HFpEF.

In this process we did note 55 cases with a documented LVEF <40% and 16 cases with a qualitative mention of ‘reduced’ LVEF. However, these cases were not excluded from the cohort as the primary method of identification for this study remained ICD code-based. The notes assessment was primarily for the purpose of validation of the ICD code-based diagnosis. Hope this clarifies the authors’ query.

*For vital signs and specific lab values first entry on the day of admission were used. In a clinical perspective using variables in a clinical model the last captured measurements before discharge may be more relevant.*

Response: Thank you for this question. We used the vitals at admission for risk modeling as our goal was to develop a risk model that could be used at admission or in the ED, especially so for the short-term (30-day) outcome. However, for the longer-term outcome (1 year) one could potentially use either the admission values or discharge values based on when the prognostication needs to be done. For an acute hospitalization there is some utility of using admission values, such as in the well-known GRACE model for acute coronary syndrome which predicts 6 month mortality and inputs admission vitals. However, the Reviewer’s suggestion is also very valid that for longer term outcomes a model at discharge has additional utility in prognostication for patients being discharged. Our model does not fill that gap and future work could focus on a discharge-specific model for HFpEF for long-term prognostication.

*Logarithmic transformations were used for certain “variables with wide ranges”. Also for variables with non-normal distribution?*

Response: Thank you for the comment regarding variable transformations. We initially referred to “logarithmic transformations” in a broad sense, however, our actual preprocessing pipeline uses power and quantile transformations to handle skewed and wide-ranging variables, thereby improving the performance of models sensitive to feature scaling (Elastic Net, Lasso, Logistic Regression, SVC, and XGBoost). Random Forest and HistGradientBoosting were trained on the original features, as tree-based methods are robust to scaling. Specifically, we applied PowerTransformer (Yeo–Johnson) and QuantileTransformer (normal output) to variables such as creatinine, INR, platelet count, WBC count, and oxygen saturation, and standardized BMI to zero mean and unit variance.

*Line 173 states gender was one-hot encoded – do authors refer to sex (binary)? Gender consists of >25 different categories.*

Response: Thank you for pointing this error out. MIMIC records the variable sex. We have made corrections to reflect this in the text as well as Table 1.  *Consider including eGFR in models instead of creatinine*

Response: Thank you for this suggestion. Unfortunately with the depth of analysis that has gone into model development any change of variable entails a re-do of the entire analysis including re-calibration of the models. Other models in the HF domain such as the Get With The Guidelines (GWTG) model for HFrEF (https://pubmed.ncbi.nlm.nih.gov/24621877/) and the PREDICT-HFpEF model in HFpEF which used the DELIVER trial data (https://pubmed.ncbi.nlm.nih.gov/38536153/) also used serum creatinine as a predictor similar to us. We hope the Reviewer is OK if we keep the current model with use of Creatinine instead of eGFR given scope of work needed to change this.
 *Results:

Table 1
How was renal disease defined? ICD codes or per eGFR?*

Response: Pre-existing renal disease in Table 1 was identified using ICD codes. Among the 36 the candidate feature as listed in Supplementary Table 3 both Prior Renal disease as identified by ICD codes as well as admission Creatinine values were used in model development.

*Treatment should be of interest to include in Table 1, even if not included in models.*

We were unsure what the Reviewer meant by ‘treatments’.

In terms of pre-hospital medications, this information is only available for 40.68% of the cohort through the medication reconciliation files.

The in-hospital treatments are available in detail only for those admissions that were to the ICU (17.65%).

Discharge medication list is available for the entire cohort who was discharged alive and we have added this to Table 1. *Fig 1, 2 and S2
Are in low solution and hard to read due to the small font.*

Response: Thank you for pointing this out. We have generated higher resolution figures which accompany the revised manuscript.  *Discussion:
A discussion on how this kind of models can be used in clinical practice would add to the manuscript.*

Response: Thank you for the comment. We have added the potential uses of our model in the discussion with the following lines:

‘The potential uses of these models could be early risk stratification for in-hospital planning such as ICU triage, and triggering care pathways like advanced HF team involvement, palliative care involvement etc. There is also a role for quality metrics and for hospitals to track post-discharge outcomes.’

Thank you again for the opportunity to submit our research work to *PLOS One*. We hope these responses address the Reviewer’s concerns. We look forward to hearing from you.

Sincerely,

Karthik Murugiah, MD

Assistant Professor of Medicine, Yale School of Medicine

Investigator, Center for Outcomes Research and Evaluation

195 Church St. 6th Floor

New Haven, CT 06510
