# Supplementary material for: Predicting 30-day and 1-year mortality in heart failure with preserved ejection fraction (HFpEF)
Source: PLoS One. 2025 Nov 14;20(11):e0336809. doi: 10.1371/journal.pone.0336809 (PMC12617840; doi:10.1371/journal.pone.0336809)
Supplement: Attachment — Submitted filename: Response_letter_R2.docx [file pone.0336809.s011.docx]

*Response to Reviewers:*

*Reviewer #1: Thank you for the nicely revised manuscript. Many of the suggested changed/clarifications have been responded to, however not implemented in the ms. Clarifications below expressed as a response to reviewer may be included in the manuscript. Difficult to track in the manuscript as they are not highlighted.*

We are happy that the Reviewer found our responses satisfactory. We apologize that we did not make more detailed additions to the manuscript. In this manuscript version we have attempted to add in some more of the response letter content and highlighted the new changes appropriately.

*Major comment and Clinical utility:*

*Can be questioned if this information adds to the clinicians’ decision and risk assessment beyond what we already know.*

*– Has to some extent been addressed in the discussion the revised ms.*

Thank you for re-emphasizing this point. We made some additions in this version addressing this point --

“Overall, in terms of the individual risk variables most are not novel such as age and NT-proBNP and have been noted in prior studies. However, the novelty of our study remains the use of real-world EHR data from a large health system to derive the model and the being model specific to the hospitalized population. In contrast prior predictive models for HFpEF have been derived from registry or trial data and are for ambulatory populations. Thus, although there is some commonality of variables identified with prior models, the strength of association with the outcomes may differ in this treatment setting. Further, the incomplete nature of EHR data also can change the association of variables. An EHR derived model is likely to perform better in a patient-facing EHR setting. However, despite these advantages further validation of our model is needed.”

*Specific points:*

*Methods:*

*Regarding the selection of patients based on ICD codes. Were patients with recorded LVEF =<40% in stable condition or during a later hospitalization excluded?*

*-Thank you for the authors response and clarification which should be added in the ms.*

Thank you for this comment. We have added additional text addressing this point --

“Given that among the 77% encounters with either a quantitative or qualitative mention of ventricular mention, >97% had a documented preserved LVEF we concluded that this method of ICD code-based identification of HFpEF is valid and has sufficient positive predictive value. This validation exercise will also be useful for future projects by other researchers that use ICD codes to identify HFpEF. In this process we did note 55 cases with a documented LVEF <40% and 16 cases with a qualitative mention of ‘reduced’ LVEF. However, these cases were not excluded from the cohort as the primary method of identification for this study remained ICD code-based. The notes assessment was primarily for the purpose of validation of the ICD code-based diagnosis.”

*-Pending: Was data extracted for the full time period 2008-2019?*

Yes, correct. To clarify again these are admissions with a primary diagnosis of HFpEF. Of 430,852 total hospitalizations in MIMIC-IV data we identified 3,235 hospitalizations with a primary diagnosis of HFpEF. This prevalence is consistent with national U.S data. Around 3% of all U.S hospitalizations are for a primary diagnosis of HF. HFpEF accounts for 30-40% of all HF (ref). Secondary diagnosis codes were not used for case identification. Thus cases of HFpEF occurring with a primary diagnosis coded as pneumonia or COPD exacerbation are not counted.

We have added this time period in the study population paragraph so this is clear.

“From a total of 430,852 hospitalizations we identified 3,235 individual hospitalization encounters with a discharge diagnosis of HFpEF which comprised the study sample.”

Ref: Russo, C. A. (Thomson Medstat), Ho, K. (AHRQ), and Elixhauser, A.(AHRQ). Hospital Stays for Circulatory Diseases, 2004. HCUP Statistical Brief #26. February 2007.

*Logarithmic transformations were used for certain “variables with wide ranges”. Also for variables with non-normal distribution?*

*-Thank you for the authors response and clarification which should be added in the ms.*

Thank you for this comment. We did incorporate the specific details of the preprocessing pipeline in our last revision, however, we apologize that they were not appropriately marked as a tracked change. We have now clearly marked this in the tracked changes version. We have also made minor additions for clarification and detail --

“Based on visual inspection of the variable distributions, we applied PowerTransformer (Yeo–Johnson) or QuantileTransformer (normal output) transformations to variables with wide ranges or evident non-normal distributions (e.g., creatinine, INR, platelet count, WBC count, and oxygen saturation), while BMI was standardized to zero mean and unit variance.”

*Results:*

*Table 1*

*How was renal disease defined? ICD codes or per eGFR?*

*-Thank you for the authors response and clarification which should be added in the ms.*

Thank you for your comment. We agree that it is helpful to clarify this in the manuscript. As we mentioned previously, among the 36 candidate features in Supplementary Table 3 both Prior Renal disease as identified by ICD codes as well as admission Creatinine values were used in model development. Certainly prior renal disease affects admission creatinine, but there is additional acuity information in admission creatinine, and thus we included both as candidate variables.

To clarify this we have added the following to the ‘Methods’ section ‘Feature Selection’ paragraph --

“Note that prior Renal disease as identified by ICD codes and admission Creatinine values were both used as individual variables in model development.”

*Treatment should be of interest to include in Table 1, even if not included in models.*

*-Thank you for adding treatment at discharge. Depending on data extraction period SGLT2i may be added.*

The use of SGLT2 inhibitors was very low in the study cohort, likely as it precedes the approval of SGLT2 inhibitors for HFpEF (FDA approval in February 2022 for empagliflozin). Thus, if the Reviewer approves, we would prefer to omit this from Table 1.

*Fig 1, 2 and S2*

*-Pending: Are in low solution and hard to read due to the small font.*

We apologize for this issue. We have generated figures 1 and 2 again at higher resolution. For figure S2 we removed the variables which have mutual information scores of zero to improve readability and increased the resolution. Hopefully they meet the journal standards now.
